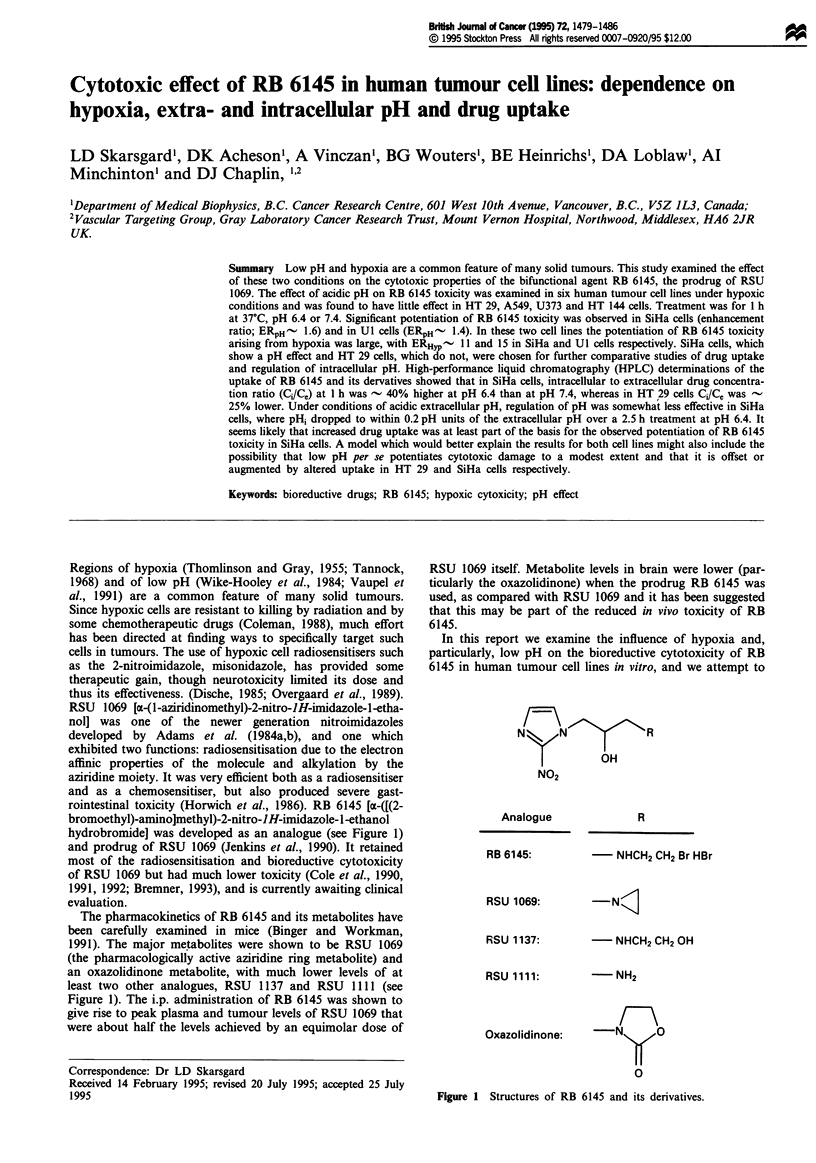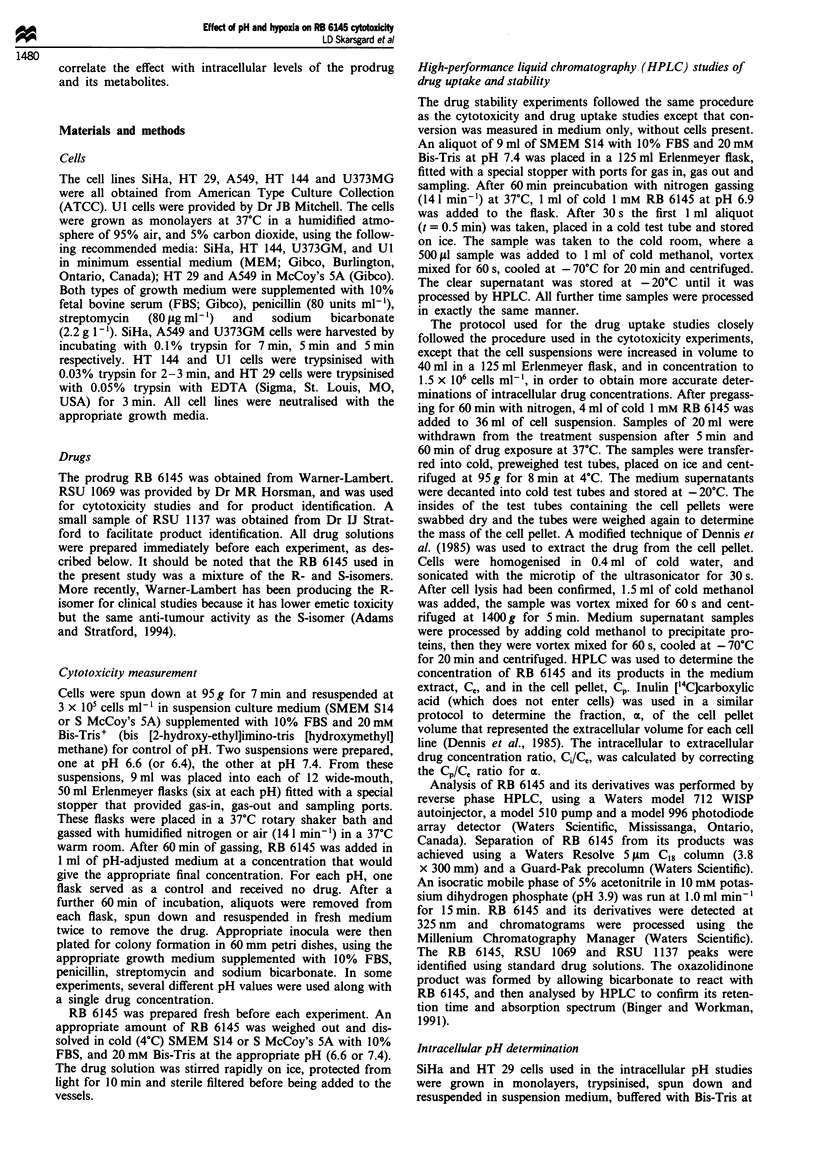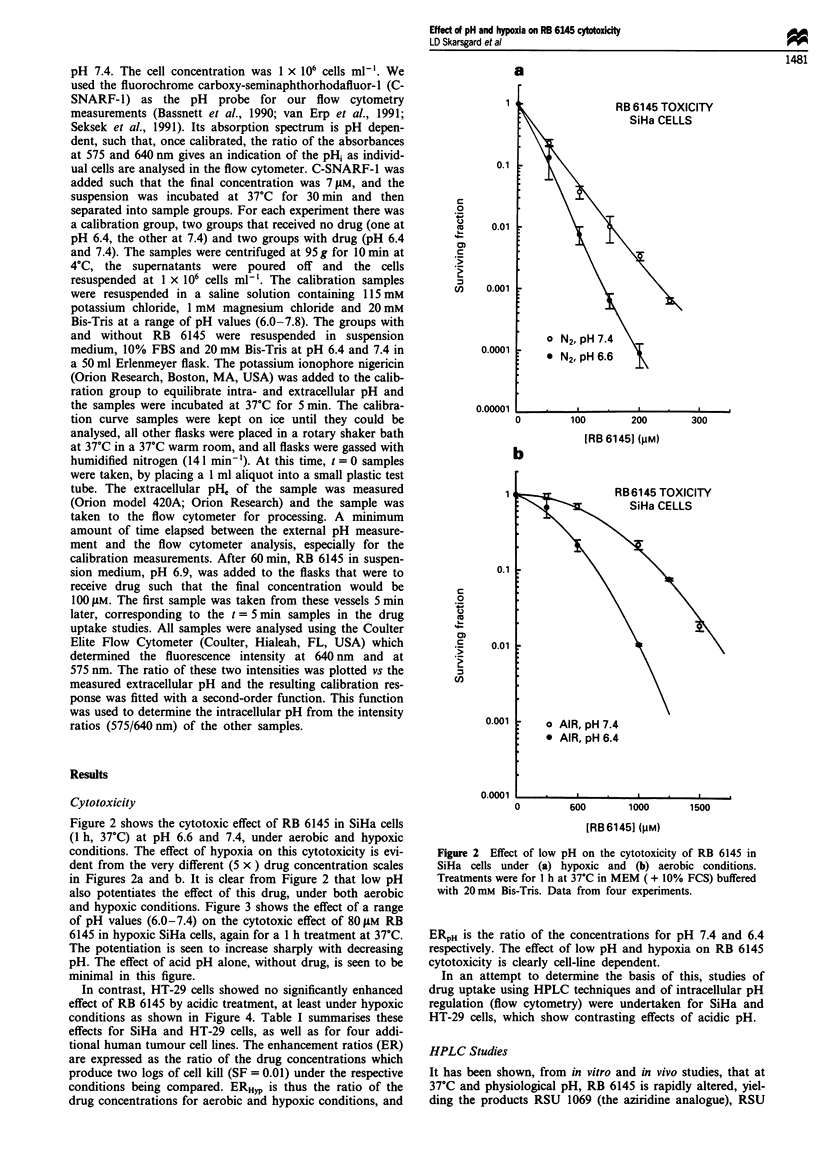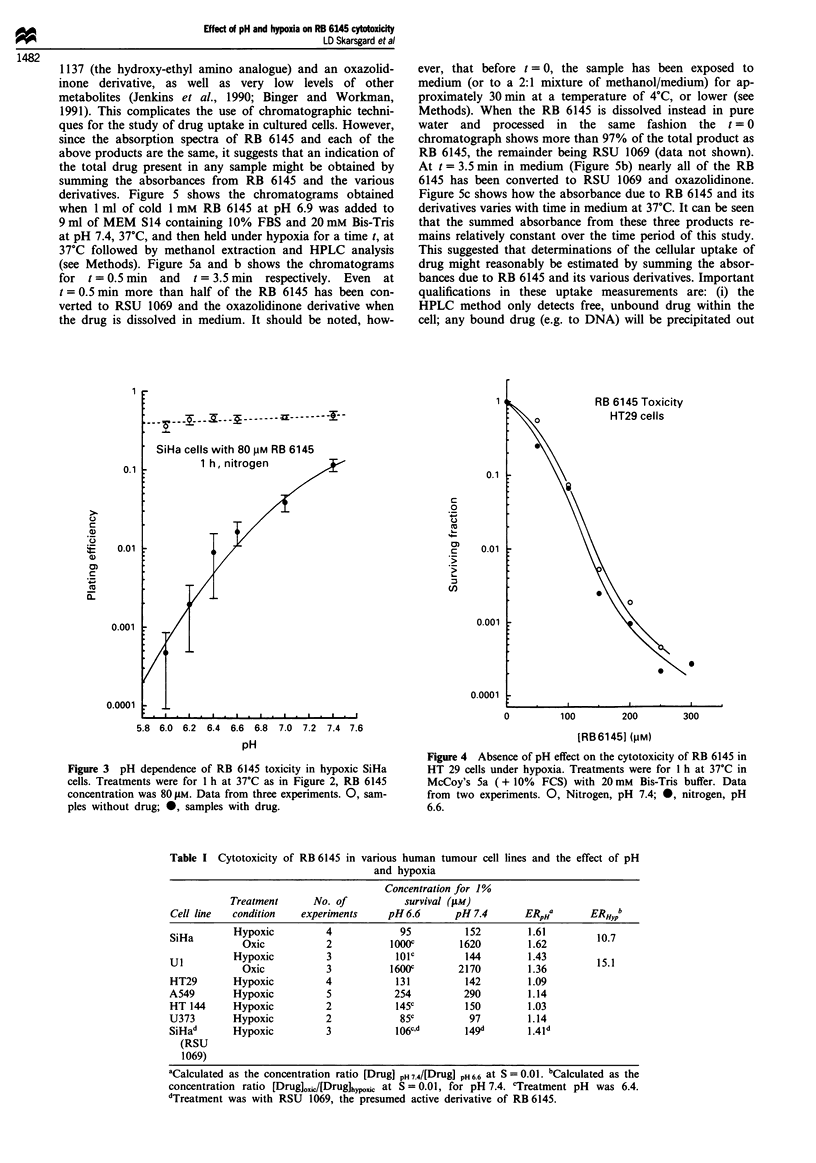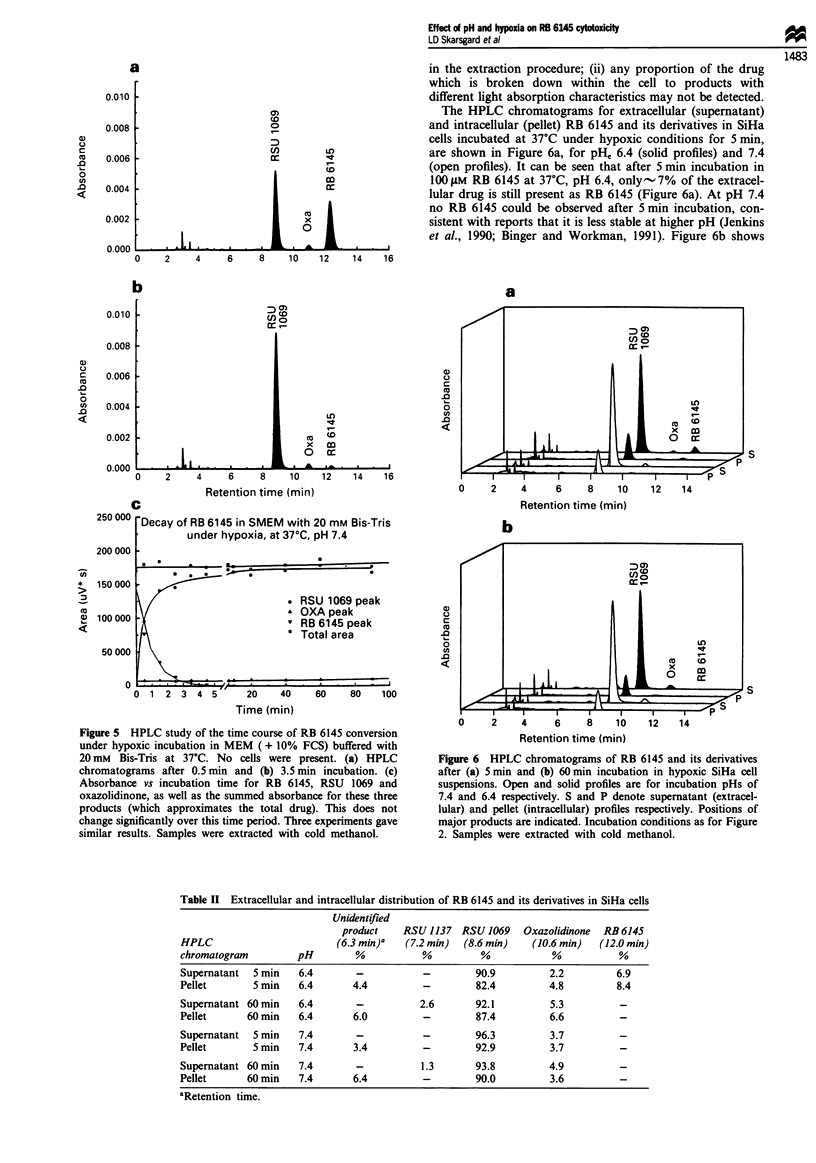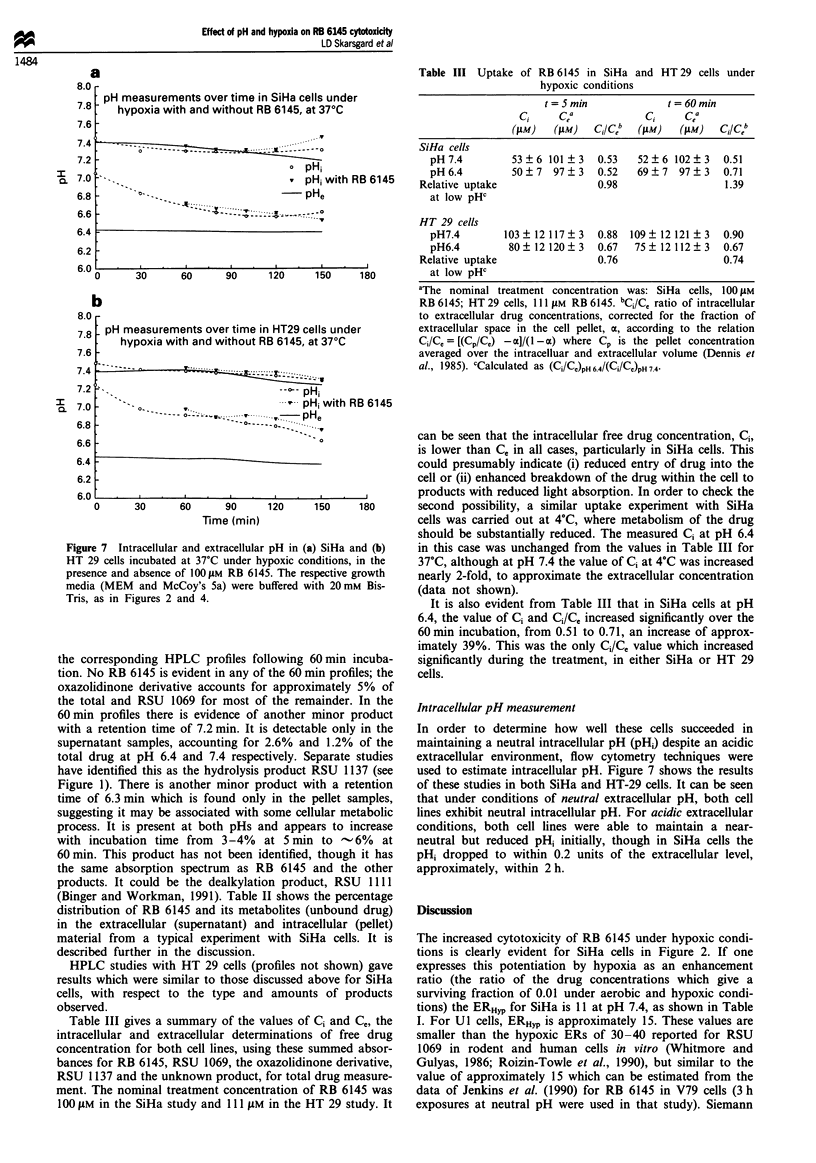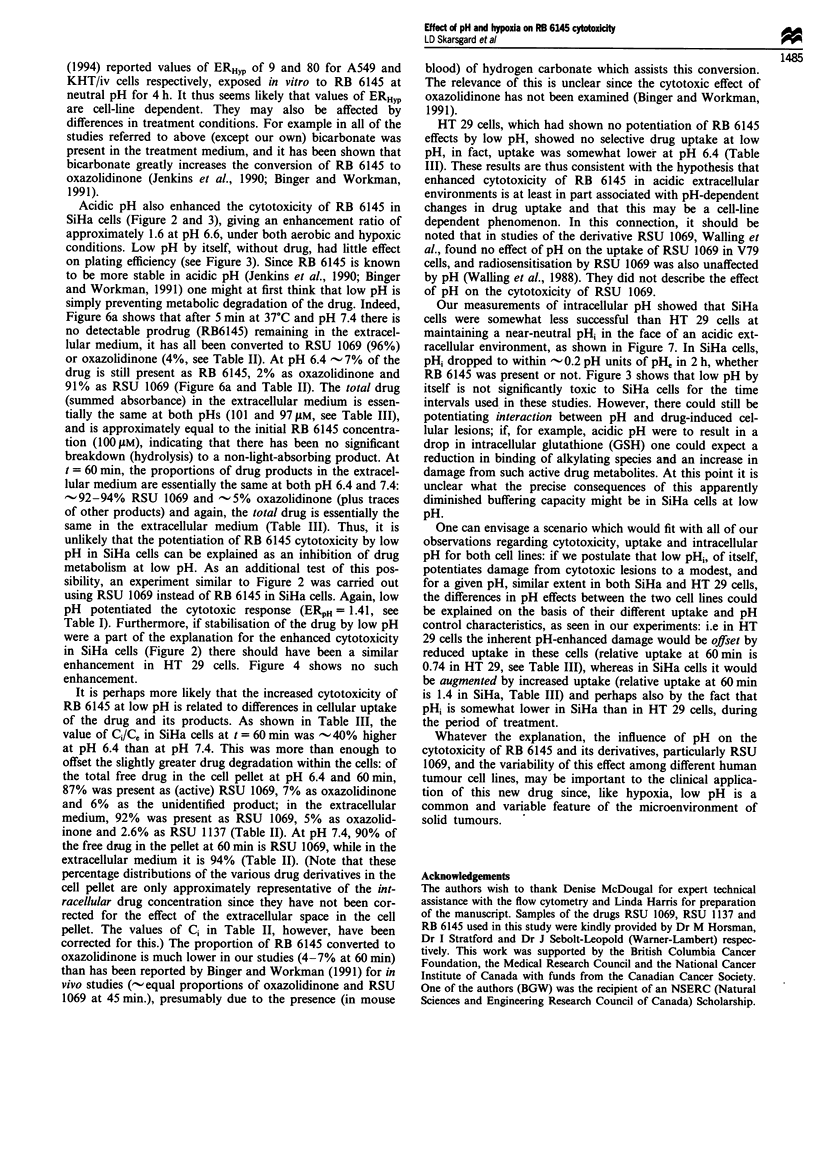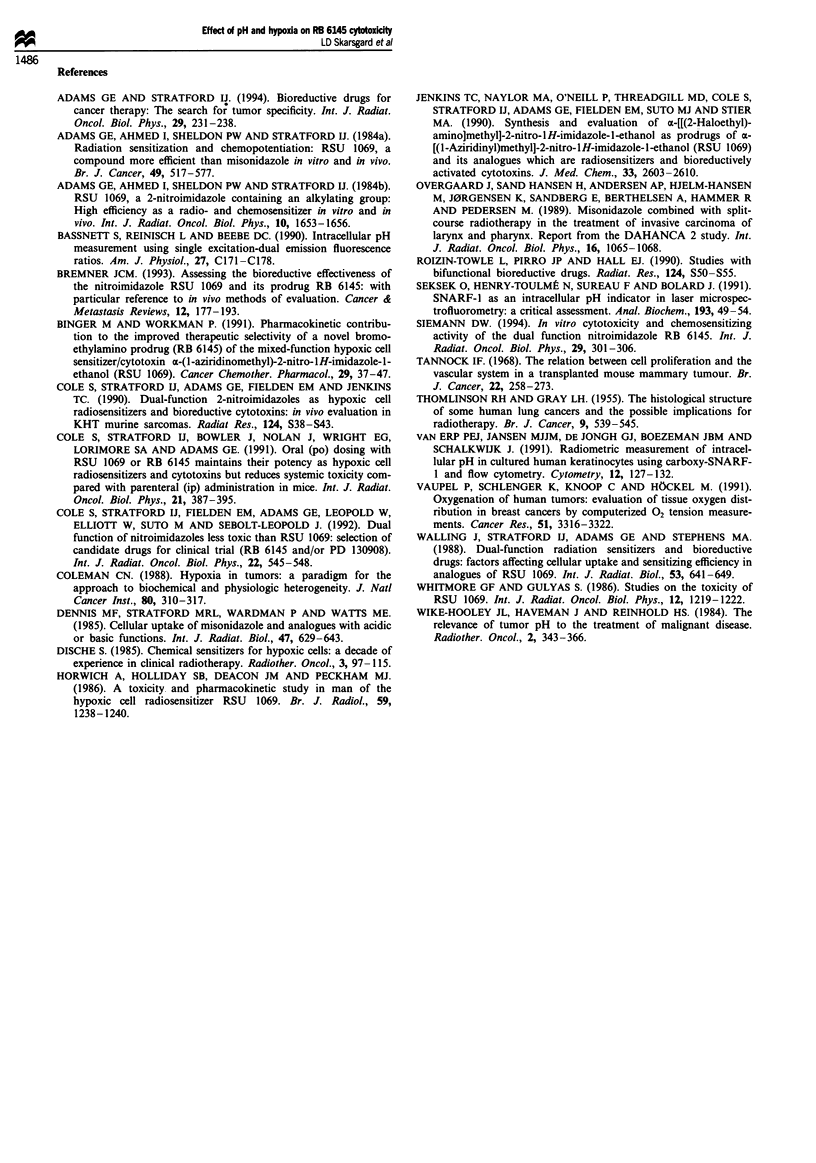# Cytotoxic effect of RB 6145 in human tumour cell lines: dependence on hypoxia, extra- and intracellular pH and drug uptake.

**DOI:** 10.1038/bjc.1995.533

**Published:** 1995-12

**Authors:** L. D. Skarsgard, D. K. Acheson, A. Vinczan, B. G. Wouters, B. E. Heinrichs, D. A. Loblaw, A. I. Minchinton, D. J. Chaplin

**Affiliations:** Department of Medical Biophysics, B.C. Cancer Research Centre, Vancouver, Canada.

## Abstract

Low pH and hypoxia are a common feature of many solid tumours. This study examined the effect of these two conditions on the cytotoxic properties of the bifunctional agent RB 6145, the prodrug of RSU 1069. The effect of acidic pH on RB 6145 toxicity was examined in six human tumour cell lines under hypoxic conditions and was found to have little effect in HT 29, A549, U373 and HT 144 cells. Treatment was for 1 h at 37 degrees C, pH 6.4 or 7.4. Significant potentiation of RB 6145 toxicity was observed in SiHa cells (enhancement ratio; ERpH approximately 1.6) and in U1 cells (ERpH approximately 1.4). In these two cell lines the potentiation of RB 6145 toxicity arising from hypoxia was large, with ERHyp approximately 11 and 15 in SiHa and U1 cells respectively. SiHa cells, which show a pH effect and HT 29 cells, which do not, were chosen for further comparative studies of drug uptake )nd regulation of intracellular pH. High-performance liquid chromatography (HPLC) determinations of the uptake of RB 6145 and its dervatives showed that in SiHa cells, intracellular to extracellular drug concentration ratio (Ci/Ce) at 1 h was approximately 40% higher at pH 6.4 than at pH 7.4, whereas in HT 29 cells Ci/Ce was approximately 25% lower. Under conditions of acidic extracellular pH, regulation of pH was somewhat less effective in SiHa cells, where pHi dropped to within 0.2 pH units of the extracellular pH over a 2.5 h treatment at pH 6.4. It seems likely that increased drug uptake was at least part of the basis for the observed potentiation of RB 6145 toxicity in SiHa cells. A model which would better explain the results for both cell lines might also include the possibility that low pH per se potentiates cytotoxic damage to a modest extent and that it is offset or augmented by altered uptake in HT 29 and SiHa cells respectively.


British Journal of Cancer (1995) 72, 1479-1486
© 1995 Stockton Press All rights reserved 0007-0920/95 $12.00

### Cytotoxic effect of RB 6145 in human tumour cell lines: dependence on hypoxia, extra- and intracellular pH and drug uptake

LD Skarsgard[1], DK Acheson[1], A Vinczan[1], BG Wouters[1], BE Heinrichs[1], DA Loblaw[1], AI Minchinton[1] and DJ Chaplin, [1,2]

[1]*Department of Medical Biophysics, B.C. Cancer Research Centre, 601 West 10th Avenue, Vancouver, B.C., V5Z 1L3, Canada;* [2]*Vascular Targeting Group, Gray Laboratory Cancer Research Trust, Mount Vernon Hospital, Northwood, Middlesex, HA6 2JR UK.*

**Summary** Low pH and hypoxia are a common feature of many solid tumours. This study examined the effect of these two conditions on the cytotoxic properties of the bifunctional agent RB 6145, the prodrug of RSU 1069. The effect of acidic pH on RB 6145 toxicity was examined in six human tumour cell lines under hypoxic conditions and was found to have little effect in HT 29, A549, U373 and HT 144 cells. Treatment was for 1 h at 37°C, pH 6.4 or 7.4. Significant potentiation of RB 6145 toxicity was observed in SiHa cells (enhancement ratio; $ER_{pH}\sim$ 1.6) and in U1 cells ($ER_{pH}\sim$ 1.4). In these two cell lines the potentiation of RB 6145 toxicity arising from hypoxia was large, with $ER_{Hyp}\sim$ 11 and 15 in SiHa and U1 cells respectively. SiHa cells, which show a pH effect and HT 29 cells, which do not, were chosen for further comparative studies of drug uptake and regulation of intracellular pH. High-performance liquid chromatography (HPLC) determinations of the uptake of RB 6145 and its dervatives showed that in SiHa cells, intracellular to extracellular drug concentration ratio ($C_i/C_e$) at 1 h was $\sim$ 40% higher at pH 6.4 than at pH 7.4, whereas in HT 29 cells $C_i/C_e$ was $\sim$ 25% lower. Under conditions of acidic extracellular pH, regulation of pH was somewhat less effective in SiHa cells, where $pH_i$ dropped to within 0.2 pH units of the extracellular pH over a 2.5 h treatment at pH 6.4. It seems likely that increased drug uptake was at least part of the basis for the observed potentiation of RB 6145 toxicity in SiHa cells. A model which would better explain the results for both cell lines might also include the possibility that low pH *per se* potentiates cytotoxic damage to a modest extent and that it is offset or augmented by altered uptake in HT 29 and SiHa cells respectively.

**Keywords:** bioreductive drugs; RB 6145; hypoxic cytoxicity; pH effect

Regions of hypoxia (Thomlinson and Gray, 1955; Tannock, 1968) and of low pH (Wike-Hooley *et al.*, 1984; Vaupel *et al.*, 1991) are a common feature of many solid tumours. Since hypoxic cells are resistant to killing by radiation and by some chemotherapeutic drugs (Coleman, 1988), much effort has been directed at finding ways to specifically target such cells in tumours. The use of hypoxic cell radiosensitisers such as the 2-nitroimidazole, misonidazole, has provided some therapeutic gain, though neurotoxicity limited its dose and thus its effectiveness. (Dische, 1985; Overgaard *et al.*, 1989). RSU 1069 [α-(1-aziridinomethyl)-2-nitro-*1H*-imidazole-1-ethanol] was one of the newer generation nitroimidazoles developed by Adams *et al.* (1984a,b), and one which exhibited two functions: radiosensitisation due to the electron affinic properties of the molecule and alkylation by the aziridine moiety. It was very efficient both as a radiosensitiser and as a chemosensitiser, but also produced severe gastrointestinal toxicity (Horwich *et al.*, 1986). RB 6145 [α-([(2-bromoethyl)-amino]methyl)-2-nitro-*1H*-imidazole-1-ethanol hydrobromide] was developed as an analogue (see Figure 1) and prodrug of RSU 1069 (Jenkins *et al.*, 1990). It retained most of the radiosensitisation and bioreductive cytotoxicity of RSU 1069 but had much lower toxicity (Cole *et al.*, 1990, 1991, 1992; Bremner, 1993), and is currently awaiting clinical evaluation.

The pharmacokinetics of RB 6145 and its metabolites have been carefully examined in mice (Binger and Workman, 1991). The major metabolites were shown to be RSU 1069 (the pharmacologically active aziridine ring metabolite) and an oxazolidinone metabolite, with much lower levels of at least two other analogues, RSU 1137 and RSU 1111 (see Figure 1). The i.p. administration of RB 6145 was shown to give rise to peak plasma and tumour levels of RSU 1069 that were about half the levels achieved by an equimolar dose of RSU 1069 itself. Metabolite levels in brain were lower (particularly the oxazolidinone) when the prodrug RB 6145 was used, as compared with RSU 1069 and it has been suggested that this may be part of the reduced *in vivo* toxicity of RB 6145.

In this report we examine the influence of hypoxia and, particularly, low pH on the bioreductive cytotoxicity of RB 6145 in human tumour cell lines *in vitro*, and we attempt to

**Figure 1** Structures of RB 6145 and its derivatives.

Correspondence: Dr LD Skarsgard
Received 14 February 1995; revised 20 July 1995; accepted 25 July 1995

correlate the effect with intracellular levels of the prodrug and its metabolites.

### Materials and methods

### Cells

The cell lines SiHa, HT 29, A549, HT 144 and U373MG were all obtained from American Type Culture Collection (ATCC). U1 cells were provided by Dr JB Mitchell. The cells were grown as monolayers at 37°C in a humidified atmosphere of 95% air, and 5% carbon dioxide, using the following recommended media: SiHa, HT 144, U373GM, and U1 in minimum essential medium (MEM; Gibco, Burlington, Ontario, Canada); HT 29 and A549 in McCoy's 5A (Gibco). Both types of growth medium were supplemented with 10% fetal bovine serum (FBS; Gibco), penicillin (80 units $ml^{-1}$), streptomycin (80 $\mu g\ ml^{-1}$) and sodium bicarbonate (2.2 g $l^{-1}$). SiHa, A549 and U373GM cells were harvested by incubating with 0.1% trypsin for 7 min, 5 min and 5 min respectively. HT 144 and U1 cells were trypsinised with 0.03% trypsin for 2–3 min, and HT 29 cells were trypsinised with 0.05% trypsin with EDTA (Sigma, St. Louis, MO, USA) for 3 min. All cell lines were neutralised with the appropriate growth media.

### Drugs

The prodrug RB 6145 was obtained from Warner-Lambert. RSU 1069 was provided by Dr MR Horsman, and was used for cytotoxicity studies and for product identification. A small sample of RSU 1137 was obtained from Dr IJ Stratford to facilitate product identification. All drug solutions were prepared immediately before each experiment, as described below. It should be noted that the RB 6145 used in the present study was a mixture of the R- and S-isomers. More recently, Warner-Lambert has been producing the R-isomer for clinical studies because it has lower emetic toxicity but the same anti-tumour activity as the S-isomer (Adams and Stratford, 1994).

### Cytotoxicity measurement

Cells were spun down at 95 *g* for 7 min and resuspended at $3 \times 10^5$ cells $ml^{-1}$ in suspension culture medium (SMEM S14 or S McCoy's 5A) supplemented with 10% FBS and 20 mM Bis-Tris$^+$ (bis [2-hydroxy-ethyl]imino-tris [hydroxymethyl] methane) for control of pH. Two suspensions were prepared, one at pH 6.6 (or 6.4), the other at pH 7.4. From these suspensions, 9 ml was placed into each of 12 wide-mouth, 50 ml Erlenmeyer flasks (six at each pH) fitted with a special stopper that provided gas-in, gas-out and sampling ports. These flasks were placed in a 37°C rotary shaker bath and gassed with humidified nitrogen or air (14 l $min^{-1}$) in a 37°C warm room. After 60 min of gassing, RB 6145 was added in 1 ml of pH-adjusted medium at a concentration that would give the appropriate final concentration. For each pH, one flask served as a control and received no drug. After a further 60 min of incubation, aliquots were removed from each flask, spun down and resuspended in fresh medium twice to remove the drug. Appropriate inocula were then plated for colony formation in 60 mm petri dishes, using the appropriate growth medium supplemented with 10% FBS, penicillin, streptomycin and sodium bicarbonate. In some experiments, several different pH values were used along with a single drug concentration.

RB 6145 was prepared fresh before each experiment. An appropriate amount of RB 6145 was weighed out and dissolved in cold (4°C) SMEM S14 or S McCoy's 5A with 10% FBS, and 20 mM Bis-Tris at the appropriate pH (6.6 or 7.4). The drug solution was stirred rapidly on ice, protected from light for 10 min and sterile filtered before being added to the vessels.

### High-performance liquid chromatography (HPLC) studies of drug uptake and stability

The drug stability experiments followed the same procedure as the cytotoxicity and drug uptake studies except that conversion was measured in medium only, without cells present. An aliquot of 9 ml of SMEM S14 with 10% FBS and 20 mM Bis-Tris at pH 7.4 was placed in a 125 ml Erlenmeyer flask, fitted with a special stopper with ports for gas in, gas out and sampling. After 60 min preincubation with nitrogen gassing (14 l $min^{-1}$) at 37°C, 1 ml of cold 1 mM RB 6145 at pH 6.9 was added to the flask. After 30 s the first 1 ml aliquot ($t = 0.5$ min) was taken, placed in a cold test tube and stored on ice. The sample was taken to the cold room, where a 500 $\mu l$ sample was added to 1 ml of cold methanol, vortex mixed for 60 s, cooled at −70°C for 20 min and centrifuged. The clear supernatant was stored at −20°C until it was processed by HPLC. All further time samples were processed in exactly the same manner.

The protocol used for the drug uptake studies closely followed the procedure used in the cytotoxicity experiments, except that the cell suspensions were increased in volume to 40 ml in a 125 ml Erlenmeyer flask, and in concentration to $1.5 \times 10^6$ cells $ml^{-1}$, in order to obtain more accurate determinations of intracellular drug concentrations. After pregassing for 60 min with nitrogen, 4 ml of cold 1 mM RB 6145 was added to 36 ml of cell suspension. Samples of 20 ml were withdrawn from the treatment suspension after 5 min and 60 min of drug exposure at 37°C. The samples were transferred into cold, preweighed test tubes, placed on ice and centrifuged at 95 *g* for 8 min at 4°C. The medium supernatants were decanted into cold test tubes and stored at −20°C. The insides of the test tubes containing the cell pellets were swabbed dry and the tubes were weighed again to determine the mass of the cell pellet. A modified technique of Dennis *et al.* (1985) was used to extract the drug from the cell pellet. Cells were homogenised in 0.4 ml of cold water, and sonicated with the microtip of the ultrasonicator for 30 s. After cell lysis had been confirmed, 1.5 ml of cold methanol was added, the sample was vortex mixed for 60 s and centrifuged at 1400 *g* for 5 min. Medium supernatant samples were processed by adding cold methanol to precipitate proteins, then they were vortex mixed for 60 s, cooled at −70°C for 20 min and centrifuged. HPLC was used to determine the concentration of RB 6145 and its products in the medium extract, $C_e$, and in the cell pellet, $C_p$. Inulin [$^{14}$C]carboxylic acid (which does not enter cells) was used in a similar protocol to determine the fraction, $\alpha$, of the cell pellet volume that represented the extracellular volume for each cell line (Dennis *et al.*, 1985). The intracellular to extracellular drug concentration ratio, $C_i/C_e$, was calculated by correcting the $C_p/C_e$ ratio for $\alpha$.

Analysis of RB 6145 and its derivatives was performed by reverse phase HPLC, using a Waters model 712 WISP autoinjector, a model 510 pump and a model 996 photodiode array detector (Waters Scientific, Mississanga, Ontario, Canada). Separation of RB 6145 from its products was achieved using a Waters Resolve 5 $\mu m$ $C_{18}$ column (3.8 × 300 mm) and a Guard-Pak precolumn (Waters Scientific). An isocratic mobile phase of 5% acetonitrile in 10 mM potassium dihydrogen phosphate (pH 3.9) was run at 1.0 ml $min^{-1}$ for 15 min. RB 6145 and its derivatives were detected at 325 nm and chromatograms were processed using the Millenium Chromatography Manager (Waters Scientific). The RB 6145, RSU 1069 and RSU 1137 peaks were identified using standard drug solutions. The oxazolidinone product was formed by allowing bicarbonate to react with RB 6145, and then analysed by HPLC to confirm its retention time and absorption spectrum (Binger and Workman, 1991).

### Intracellular pH determination

SiHa and HT 29 cells used in the intracellular pH studies were grown in monolayers, trypsinised, spun down and resuspended in suspension medium, buffered with Bis-Tris at pH 7.4. The cell concentration was $1 \times 10^6$ cells $ml^{-1}$. We used the fluorochrome carboxy-seminaphthorhodafluor-1 (C-SNARF-1) as the pH probe for our flow cytometry measurements (Bassnett *et al.*, 1990; van Erp *et al.*, 1991; Seksek *et al.*, 1991). Its absorption spectrum is pH dependent, such that, once calibrated, the ratio of the absorbances at 575 and 640 nm gives an indication of the $pH_i$ as individual cells are analysed in the flow cytometer. C-SNARF-1 was added such that the final concentration was 7 μM, and the suspension was incubated at 37°C for 30 min and then separated into sample groups. For each experiment there was a calibration group, two groups that received no drug (one at pH 6.4, the other at 7.4) and two groups with drug (pH 6.4 and 7.4). The samples were centrifuged at 95 *g* for 10 min at 4°C, the supernatants were poured off and the cells resuspended at $1 \times 10^6$ cells $ml^{-1}$. The calibration samples were resuspended in a saline solution containing 115 mM potassium chloride, 1 mM magnesium chloride and 20 mM Bis-Tris at a range of pH values (6.0–7.8). The groups with and without RB 6145 were resuspended in suspension medium, 10% FBS and 20 mM Bis-Tris at pH 6.4 and 7.4 in a 50 ml Erlenmeyer flask. The potassium ionophore nigericin (Orion Research, Boston, MA, USA) was added to the calibration group to equilibrate intra- and extracellular pH and the samples were incubated at 37°C for 5 min. The calibration curve samples were kept on ice until they could be analysed, all other flasks were placed in a rotary shaker bath at 37°C in a 37°C warm room, and all flasks were gassed with humidified nitrogen (14 l $min^{-1}$). At this time, $t = 0$ samples were taken, by placing a 1 ml aliquot into a small plastic test tube. The extracellular $pH_e$ of the sample was measured (Orion model 420A; Orion Research) and the sample was taken to the flow cytometer for processing. A minimum amount of time elapsed between the external pH measurement and the flow cytometer analysis, especially for the calibration measurements. After 60 min, RB 6145 in suspension medium, pH 6.9, was added to the flasks that were to receive drug such that the final concentration would be 100 μM. The first sample was taken from these vessels 5 min later, corresponding to the $t = 5$ min samples in the drug uptake studies. All samples were analysed using the Coulter Elite Flow Cytometer (Coulter, Hialeah, FL, USA) which determined the fluorescence intensity at 640 nm and at 575 nm. The ratio of these two intensities was plotted *vs* the measured extracellular pH and the resulting calibration response was fitted with a second-order function. This function was used to determine the intracellular pH from the intensity ratios (575/640 nm) of the other samples.

### Results

### *Cytotoxicity*

Figure 2 shows the cytotoxic effect of RB 6145 in SiHa cells (1 h, 37°C) at pH 6.6 and 7.4, under aerobic and hypoxic conditions. The effect of hypoxia on this cytotoxicity is evident from the very different (5 × ) drug concentration scales in Figures 2a and b. It is clear from Figure 2 that low pH also potentiates the effect of this drug, under both aerobic and hypoxic conditions. Figure 3 shows the effect of a range of pH values (6.0–7.4) on the cytotoxic effect of 80 μM RB 6145 in hypoxic SiHa cells, again for a 1 h treatment at 37°C. The potentiation is seen to increase sharply with decreasing pH. The effect of acid pH alone, without drug, is seen to be minimal in this figure.

In contrast, HT-29 cells showed no significantly enhanced effect of RB 6145 by acidic treatment, at least under hypoxic conditions as shown in Figure 4. Table I summarises these effects for SiHa and HT-29 cells, as well as for four additional human tumour cell lines. The enhancement ratios (ER) are expressed as the ratio of the drug concentrations which produce two logs of cell kill (SF = 0.01) under the respective conditions being compared. $ER_{Hyp}$ is thus the ratio of the drug concentrations for aerobic and hypoxic conditions, and

**Figure 2** Effect of low pH on the cytotoxicity of RB 6145 in SiHa cells under (**a**) hypoxic and (**b**) aerobic conditions. Treatments were for 1 h at 37°C in MEM ( + 10% FCS) buffered with 20 mM Bis-Tris. Data from four experiments.

$ER_{pH}$ is the ratio of the concentrations for pH 7.4 and 6.4 respectively. The effect of low pH and hypoxia on RB 6145 cytotoxicity is clearly cell-line dependent.

In an attempt to determine the basis of this, studies of drug uptake using HPLC techniques and of intracellular pH regulation (flow cytometry) were undertaken for SiHa and HT-29 cells, which show contrasting effects of acidic pH.

### *HPLC Studies*

It has been shown, from *in vitro* and *in vivo* studies, that at 37°C and physiological pH, RB 6145 is rapidly altered, yielding the products RSU 1069 (the aziridine analogue), RSU

1137 (the hydroxy-ethyl amino analogue) and an oxazolidinone derivative, as well as very low levels of other metabolites (Jenkins *et al.*, 1990; Binger and Workman, 1991). This complicates the use of chromatographic techniques for the study of drug uptake in cultured cells. However, since the absorption spectra of RB 6145 and each of the above products are the same, it suggests that an indication of the total drug present in any sample might be obtained by summing the absorbances from RB 6145 and the various derivatives. Figure 5 shows the chromatograms obtained when 1 ml of cold 1 mM RB 6145 at pH 6.9 was added to 9 ml of MEM S14 containing 10% FBS and 20 mM Bis-Tris at pH 7.4, 37°C, and then held under hypoxia for a time $t$, at 37°C followed by methanol extraction and HPLC analysis (see Methods). Figure 5a and b shows the chromatograms for $t = 0.5$ min and $t = 3.5$ min respectively. Even at $t = 0.5$ min more than half of the RB 6145 has been converted to RSU 1069 and the oxazolidinone derivative when the drug is dissolved in medium. It should be noted, however, that before $t = 0$, the sample has been exposed to medium (or to a 2:1 mixture of methanol/medium) for approximately 30 min at a temperature of 4°C, or lower (see Methods). When the RB 6145 is dissolved instead in pure water and processed in the same fashion the $t = 0$ chromatograph shows more than 97% of the total product as RB 6145, the remainder being RSU 1069 (data not shown). At $t = 3.5$ min in medium (Figure 5b) nearly all of the RB 6145 has been converted to RSU 1069 and oxazolidinone. Figure 5c shows how the absorbance due to RB 6145 and its derivatives varies with time in medium at 37°C. It can be seen that the summed absorbance from these three products remains relatively constant over the time period of this study. This suggested that determinations of the cellular uptake of drug might reasonably be estimated by summing the absorbances due to RB 6145 and its various derivatives. Important qualifications in these uptake measurements are: (i) the HPLC method only detects free, unbound drug within the cell; any bound drug (e.g. to DNA) will be precipitated out

**Figure 3** pH dependence of RB 6145 toxicity in hypoxic SiHa cells. Treatments were for 1 h at 37°C as in Figure 2, RB 6145 concentration was 80 μM. Data from three experiments. O, samples without drug; ●, samples with drug.

**Figure 4** Absence of pH effect on the cytotoxicity of RB 6145 in HT 29 cells under hypoxia. Treatments were for 1 h at 37°C in McCoy's 5a ( + 10% FCS) with 20 mM Bis-Tris buffer. Data from two experiments. O, Nitrogen, pH 7.4; ●, nitrogen, pH 6.6.

**Table I** Cytotoxicity of RB 6145 in various human tumour cell lines and the effect of pH and hypoxia

| *Cell line* | *Treatment condition* | *No. of experiments* | *Concentration for 1% survival (μM)* *pH 6.6* | *pH 7.4* | $ER_{pH}{}^a$ | $ER_{Hyp}{}^b$ |
|---|---|---|---|---|---|---|
| SiHa | Hypoxic | 4 | 95 | 152 | 1.61 | 10.7 |
| | Oxic | 2 | 1000[c] | 1620 | 1.62 | |
| U1 | Hypoxic | 3 | 101[c] | 144 | 1.43 | 15.1 |
| | Oxic | 3 | 1600[c] | 2170 | 1.36 | |
| HT29 | Hypoxic | 4 | 131 | 142 | 1.09 | |
| A549 | Hypoxic | 5 | 254 | 290 | 1.14 | |
| HT 144 | Hypoxic | 2 | 145[c] | 150 | 1.03 | |
| U373 | Hypoxic | 2 | 85[c] | 97 | 1.14 | |
| SiHa[d] (RSU 1069) | Hypoxic | 3 | 106[c,d] | 149[d] | 1.41[d] | |

[a]Calculated as the concentration ratio $[Drug]_{pH\,7.4}/[Drug]_{pH\,6.6}$ at $S = 0.01$. [b]Calculated as the concentration ratio $[Drug]_{oxic}/[Drug]_{hypoxic}$ at $S = 0.01$, for pH 7.4. [c]Treatment pH was 6.4. [d]Treatment was with RSU 1069, the presumed active derivative of RB 6145.

**Figure 5** HPLC study of the time course of RB 6145 conversion under hypoxic incubation in MEM ( + 10% FCS) buffered with 20 mM Bis-Tris at 37°C. No cells were present. (**a**) HPLC chromatograms after 0.5 min and (**b**) 3.5 min incubation. (**c**) Absorbance *vs* incubation time for RB 6145, RSU 1069 and oxazolidinone, as well as the summed absorbance for these three products (which approximates the total drug). This does not change significantly over this time period. Three experiments gave similar results. Samples were extracted with cold methanol.

in the extraction procedure; (ii) any proportion of the drug which is broken down within the cell to products with different light absorption characteristics may not be detected.

The HPLC chromatograms for extracellular (supernatant) and intracellular (pellet) RB 6145 and its derivatives in SiHa cells incubated at 37°C under hypoxic conditions for 5 min, are shown in Figure 6a, for $pH_e$ 6.4 (solid profiles) and 7.4 (open profiles). It can be seen that after 5 min incubation in 100 μM RB 6145 at 37°C, pH 6.4, only ~7% of the extracellular drug is still present as RB 6145 (Figure 6a). At pH 7.4 no RB 6145 could be observed after 5 min incubation, consistent with reports that it is less stable at higher pH (Jenkins *et al.*, 1990; Binger and Workman, 1991). Figure 6b shows

**Figure 6** HPLC chromatograms of RB 6145 and its derivatives after (**a**) 5 min and (**b**) 60 min incubation in hypoxic SiHa cell suspensions. Open and solid profiles are for incubation pHs of 7.4 and 6.4 respectively. S and P denote supernatant (extracellular) and pellet (intracellular) profiles respectively. Positions of major products are indicated. Incubation conditions as for Figure 2. Samples were extracted with cold methanol.

**Table II** Extracellular and intracellular distribution of RB 6145 and its derivatives in SiHa cells

| *HPLC chromatogram* | | *pH* | *Unidentified product (6.3 min)[a] %* | *RSU 1137 (7.2 min) %* | *RSU 1069 (8.6 min) %* | *Oxazolidinone (10.6 min) %* | *RB 6145 (12.0 min) %* |
|---|---|---|---|---|---|---|---|
| Supernatant | 5 min | 6.4 | – | – | 90.9 | 2.2 | 6.9 |
| Pellet | 5 min | 6.4 | 4.4 | – | 82.4 | 4.8 | 8.4 |
| Supernatant | 60 min | 6.4 | – | 2.6 | 92.1 | 5.3 | – |
| Pellet | 60 min | 6.4 | 6.0 | – | 87.4 | 6.6 | – |
| Supernatant | 5 min | 7.4 | – | – | 96.3 | 3.7 | – |
| Pellet | 5 min | 7.4 | 3.4 | – | 92.9 | 3.7 | – |
| Supernatant | 60 min | 7.4 | – | 1.3 | 93.8 | 4.9 | – |
| Pellet | 60 min | 7.4 | 6.4 | – | 90.0 | 3.6 | – |

[a]Retention time.

**Figure 7** Intracellular and extracellular pH in (**a**) SiHa and (**b**) HT 29 cells incubated at 37°C under hypoxic conditions, in the presence and absence of 100 μM RB 6145. The respective growth media (MEM and McCoy's 5a) were buffered with 20 mM Bis-Tris, as in Figures 2 and 4.

the corresponding HPLC profiles following 60 min incubation. No RB 6145 is evident in any of the 60 min profiles; the oxazolidinone derivative accounts for approximately 5% of the total and RSU 1069 for most of the remainder. In the 60 min profiles there is evidence of another minor product with a retention time of 7.2 min. It is detectable only in the supernatant samples, accounting for 2.6% and 1.2% of the total drug at pH 6.4 and 7.4 respectively. Separate studies have identified this as the hydrolysis product RSU 1137 (see Figure 1). There is another minor product with a retention time of 6.3 min which is found only in the pellet samples, suggesting it may be associated with some cellular metabolic process. It is present at both pHs and appears to increase with incubation time from 3–4% at 5 min to ~6% at 60 min. This product has not been identified, though it has the same absorption spectrum as RB 6145 and the other products. It could be the dealkylation product, RSU 1111 (Binger and Workman, 1991). Table II shows the percentage distribution of RB 6145 and its metabolites (unbound drug) in the extracellular (supernatant) and intracellular (pellet) material from a typical experiment with SiHa cells. It is described further in the discussion.

HPLC studies with HT 29 cells (profiles not shown) gave results which were similar to those discussed above for SiHa cells, with respect to the type and amounts of products observed.

Table III gives a summary of the values of $C_i$ and $C_e$, the intracellular and extracellular determinations of free drug concentration for both cell lines, using these summed absorbances for RB 6145, RSU 1069, the oxazolidinone derivative, RSU 1137 and the unknown product, for total drug measurement. The nominal treatment concentration of RB 6145 was 100 μM in the SiHa study and 111 μM in the HT 29 study. It

**Table III** Uptake of RB 6145 in SiHa and HT 29 cells under hypoxic conditions

| | *t = 5 min* | | | *t = 60 min* | | |
|---|---|---|---|---|---|---|
| | $C_i$ (μM) | $C_e^a$ (μM) | $C_i/C_e^b$ | $C_i$ (μM) | $C_e^a$ (μM) | $C_i/C_e^b$ |
| *SiHa cells* | | | | | | |
| pH 7.4 | 53 ± 6 | 101 ± 3 | 0.53 | 52 ± 6 | 102 ± 3 | 0.51 |
| pH 6.4 | 50 ± 7 | 97 ± 3 | 0.52 | 69 ± 7 | 97 ± 3 | 0.71 |
| Relative uptake at low pH[c] | | | 0.98 | | | 1.39 |
| *HT 29 cells* | | | | | | |
| pH7.4 | 103 ± 12 | 117 ± 3 | 0.88 | 109 ± 12 | 121 ± 3 | 0.90 |
| pH6.4 | 80 ± 12 | 120 ± 3 | 0.67 | 75 ± 12 | 112 ± 3 | 0.67 |
| Relative uptake at low pH[c] | | | 0.76 | | | 0.74 |

[a]The nominal treatment concentration was: SiHa cells, 100 μM RB 6145; HT 29 cells, 111 μM RB 6145. [b]$C_i/C_e$ ratio of intracellular to extracellular drug concentrations, corrected for the fraction of extracellular space in the cell pellet, α, according to the relation $C_i/C_e = [(C_p/C_e) - \alpha]/(1-\alpha)$ where $C_p$ is the pellet concentration averaged over the intracelluar and extracellular volume (Dennis *et al.*, 1985). [c]Calculated as $(C_i/C_e)_{pH\,6.4}/(C_i/C_e)_{pH\,7.4}$.

can be seen that the intracellular free drug concentration, $C_i$, is lower than $C_e$ in all cases, particularly in SiHa cells. This could presumably indicate (i) reduced entry of drug into the cell or (ii) enhanced breakdown of the drug within the cell to products with reduced light absorption. In order to check the second possibility, a similar uptake experiment with SiHa cells was carried out at 4°C, where metabolism of the drug should be substantially reduced. The measured $C_i$ at pH 6.4 in this case was unchanged from the values in Table III for 37°C, although at pH 7.4 the value of $C_i$ at 4°C was increased nearly 2-fold, to approximate the extracellular concentration (data not shown).

It is also evident from Table III that in SiHa cells at pH 6.4, the value of $C_i$ and $C_i/C_e$ increased significantly over the 60 min incubation, from 0.51 to 0.71, an increase of approximately 39%. This was the only $C_i/C_e$ value which increased significantly during the treatment, in either SiHa or HT 29 cells.

*Intracellular pH measurement*

In order to determine how well these cells succeeded in maintaining a neutral intracellular pH ($pH_i$) despite an acidic extracellular environment, flow cytometry techniques were used to estimate intracellular pH. Figure 7 shows the results of these studies in both SiHa and HT-29 cells. It can be seen that under conditions of *neutral* extracellular pH, both cell lines exhibit neutral intracellular pH. For *acidic* extracellular conditions, both cell lines were able to maintain a near-neutral but reduced $pH_i$ initially, though in SiHa cells the $pH_i$ dropped to within 0.2 units of the extracellular level, approximately, within 2 h.

### Discussion

The increased cytotoxicity of RB 6145 under hypoxic conditions is clearly evident for SiHa cells in Figure 2. If one expresses this potentiation by hypoxia as an enhancement ratio (the ratio of the drug concentrations which give a surviving fraction of 0.01 under aerobic and hypoxic conditions) the $ER_{Hyp}$ for SiHa is 11 at pH 7.4, as shown in Table I. For U1 cells, $ER_{Hyp}$ is approximately 15. These values are smaller than the hypoxic ERs of 30–40 reported for RSU 1069 in rodent and human cells *in vitro* (Whitmore and Gulyas, 1986; Roizin-Towle *et al.*, 1990), but similar to the value of approximately 15 which can be estimated from the data of Jenkins *et al.* (1990) for RB 6145 in V79 cells (3 h exposures at neutral pH were used in that study). Siemann

(1994) reported values of $ER_{Hyp}$ of 9 and 80 for A549 and KHT/iv cells respectively, exposed *in vitro* to RB 6145 at neutral pH for 4 h. It thus seems likely that values of $ER_{Hyp}$ are cell-line dependent. They may also be affected by differences in treatment conditions. For example in all of the studies referred to above (except our own) bicarbonate was present in the treatment medium, and it has been shown that bicarbonate greatly increases the conversion of RB 6145 to oxazolidinone (Jenkins *et al.*, 1990; Binger and Workman, 1991).

Acidic pH also enhanced the cytotoxicity of RB 6145 in SiHa cells (Figure 2 and 3), giving an enhancement ratio of approximately 1.6 at pH 6.6, under both aerobic and hypoxic conditions. Low pH by itself, without drug, had little effect on plating efficiency (see Figure 3). Since RB 6145 is known to be more stable in acidic pH (Jenkins *et al.*, 1990; Binger and Workman, 1991) one might at first think that low pH is simply preventing metabolic degradation of the drug. Indeed, Figure 6a shows that after 5 min at 37°C and pH 7.4 there is no detectable prodrug (RB6145) remaining in the extracellular medium, it has all been converted to RSU 1069 (96%) or oxazolidinone (4%, see Table II). At pH 6.4 ~7% of the drug is still present as RB 6145, 2% as oxazolidinone and 91% as RSU 1069 (Figure 6a and Table II). The *total* drug (summed absorbance) in the extracellular medium is essentially the same at both pHs (101 and 97 μM, see Table III), and is approximately equal to the initial RB 6145 concentration (100 μM), indicating that there has been no significant breakdown (hydrolysis) to a non-light-absorbing product. At $t = 60$ min, the proportions of drug products in the extracellular medium are essentially the same at both pH 6.4 and 7.4: ~92–94% RSU 1069 and ~5% oxazolidinone (plus traces of other products) and again, the *total* drug is essentially the same in the extracellular medium (Table III). Thus, it is unlikely that the potentiation of RB 6145 cytotoxicity by low pH in SiHa cells can be explained as an inhibition of drug metabolism at low pH. As an additional test of this possibility, an experiment similar to Figure 2 was carried out using RSU 1069 instead of RB 6145 in SiHa cells. Again, low pH potentiated the cytotoxic response ($ER_{pH} = 1.41$, see Table I). Furthermore, if stabilisation of the drug by low pH were a part of the explanation for the enhanced cytotoxicity in SiHa cells (Figure 2) there should have been a similar enhancement in HT 29 cells. Figure 4 shows no such enhancement.

It is perhaps more likely that the increased cytotoxicity of RB 6145 at low pH is related to differences in cellular uptake of the drug and its products. As shown in Table III, the value of $C_i/C_e$ in SiHa cells at $t = 60$ min was ~40% higher at pH 6.4 than at pH 7.4. This was more than enough to offset the slightly greater drug degradation within the cells: of the total free drug in the cell pellet at pH 6.4 and 60 min, 87% was present as (active) RSU 1069, 7% as oxazolidinone and 6% as the unidentified product; in the extracellular medium, 92% was present as RSU 1069, 5% as oxazolidinone and 2.6% as RSU 1137 (Table II). At pH 7.4, 90% of the free drug in the pellet at 60 min is RSU 1069, while in the extracellular medium it is 94% (Table II). (Note that these percentage distributions of the various drug derivatives in the cell pellet are only approximately representative of the *intracellular* drug concentration since they have not been corrected for the effect of the extracellular space in the cell pellet. The values of $C_i$ in Table II, however, have been corrected for this.) The proportion of RB 6145 converted to oxazolidinone is much lower in our studies (4–7% at 60 min) than has been reported by Binger and Workman (1991) for *in vivo* studies (~equal proportions of oxazolidinone and RSU 1069 at 45 min.), presumably due to the presence (in mouse blood) of hydrogen carbonate which assists this conversion. The relevance of this is unclear since the cytotoxic effect of oxazolidinone has not been examined (Binger and Workman, 1991).

HT 29 cells, which had shown no potentiation of RB 6145 effects by low pH, showed no selective drug uptake at low pH, in fact, uptake was somewhat lower at pH 6.4 (Table III). These results are thus consistent with the hypothesis that enhanced cytotoxicity of RB 6145 in acidic extracellular environments is at least in part associated with pH-dependent changes in drug uptake and that this may be a cell-line dependent phenomenon. In this connection, it should be noted that in studies of the derivative RSU 1069, Walling *et al.*, found no effect of pH on the uptake of RSU 1069 in V79 cells, and radiosensitisation by RSU 1069 was also unaffected by pH (Walling *et al.*, 1988). They did not describe the effect of pH on the cytotoxicity of RSU 1069.

Our measurements of intracellular pH showed that SiHa cells were somewhat less successful than HT 29 cells at maintaining a near-neutral $pH_i$ in the face of an acidic extracellular environment, as shown in Figure 7. In SiHa cells, $pH_i$ dropped to within ~0.2 pH units of $pH_e$ in 2 h, whether RB 6145 was present or not. Figure 3 shows that low pH by itself is not significantly toxic to SiHa cells for the time intervals used in these studies. However, there could still be potentiating *interaction* between pH and drug-induced cellular lesions; if, for example, acidic pH were to result in a drop in intracellular glutathione (GSH) one could expect a reduction in binding of alkylating species and an increase in damage from such active drug metabolites. At this point it is unclear what the precise consequences of this apparently diminished buffering capacity might be in SiHa cells at low pH.

One can envisage a scenario which would fit with all of our observations regarding cytotoxicity, uptake and intracellular pH for both cell lines: if we postulate that low $pH_i$, of itself, potentiates damage from cytotoxic lesions to a modest, and for a given pH, similar extent in both SiHa and HT 29 cells, the differences in pH effects between the two cell lines could be explained on the basis of their different uptake and pH control characteristics, as seen in our experiments: i.e in HT 29 cells the inherent pH-enhanced damage would be *offset* by reduced uptake in these cells (relative uptake at 60 min is 0.74 in HT 29, see Table III), whereas in SiHa cells it would be *augmented* by increased uptake (relative uptake at 60 min is 1.4 in SiHa, Table III) and perhaps also by the fact that $pH_i$ is somewhat lower in SiHa than in HT 29 cells, during the period of treatment.

Whatever the explanation, the influence of pH on the cytotoxicity of RB 6145 and its derivatives, particularly RSU 1069, and the variability of this effect among different human tumour cell lines, may be important to the clinical application of this new drug since, like hypoxia, low pH is a common and variable feature of the microenvironment of solid tumours.

**Acknowledgements**

The authors wish to thank Denise McDougal for expert technical assistance with the flow cytometry and Linda Harris for preparation of the manuscript. Samples of the drugs RSU 1069, RSU 1137 and RB 6145 used in this study were kindly provided by Dr M Horsman, Dr I Stratford and Dr J Sebolt-Leopold (Warner-Lambert) respectively. This work was supported by the British Columbia Cancer Foundation, the Medical Research Council and the National Cancer Institute of Canada with funds from the Canadian Cancer Society. One of the authors (BGW) was the recipient of an NSERC (Natural Sciences and Engineering Research Council of Canada) Scholarship.

### References

ADAMS GE AND STRATFORD IJ. (1994). Bioreductive drugs for cancer therapy: The search for tumor specificity. *Int. J. Radiat. Oncol. Biol. Phys.*, **29**, 231–238.

ADAMS GE, AHMED I, SHELDON PW AND STRATFORD IJ. (1984a). Radiation sensitization and chemopotentiation: RSU 1069, a compound more efficient than misonidazole *in vitro* and *in vivo*. *Br. J. Cancer*, **49**, 517–577.

ADAMS GE, AHMED I, SHELDON PW AND STRATFORD IJ. (1984b). RSU 1069, a 2-nitroimidazole containing an alkylating group: High efficiency as a radio- and chemosensitizer *in vitro* and *in vivo*. *Int. J. Radiat. Oncol. Biol. Phys.*, **10**, 1653–1656.

BASSNETT S, REINISCH L AND BEEBE DC. (1990). Intracellular pH measurement using single excitation-dual emission fluorescence ratios. *Am. J. Physiol.*, **27**, C171–C178.

BREMNER JCM. (1993). Assessing the bioreductive effectiveness of the nitroimidazole RSU 1069 and its prodrug RB 6145: with particular reference to *in vivo* methods of evaluation. *Cancer & Metastasis Reviews*, **12**, 177–193.

BINGER M AND WORKMAN P. (1991). Pharmacokinetic contribution to the improved therapeutic selectivity of a novel bromoethylamino prodrug (RB 6145) of the mixed-function hypoxic cell sensitizer/cytotoxin α-(1-aziridinomethyl)-2-nitro-1*H*-imidazole-1-ethanol (RSU 1069). *Cancer Chemother. Pharmacol.*, **29**, 37–47.

COLE S, STRATFORD IJ, ADAMS GE, FIELDEN EM AND JENKINS TC. (1990). Dual-function 2-nitroimidazoles as hypoxic cell radiosensitizers and bioreductive cytotoxins: *in vivo* evaluation in KHT murine sarcomas. *Radiat Res.*, **124**, S38–S43.

COLE S, STRATFORD IJ, BOWLER J, NOLAN J, WRIGHT EG, LORIMORE SA AND ADAMS GE. (1991). Oral (po) dosing with RSU 1069 or RB 6145 maintains their potency as hypoxic cell radiosensitizers and cytotoxins but reduces systemic toxicity compared with parenteral (ip) administration in mice. *Int. J. Radiat. Oncol. Biol. Phys.*, **21**, 387–395.

COLE S, STRATFORD IJ, FIELDEN EM, ADAMS GE, LEOPOLD W, ELLIOTT W, SUTO M AND SEBOLT-LEOPOLD J. (1992). Dual function of nitroimidazoles less toxic than RSU 1069: selection of candidate drugs for clinical trial (RB 6145 and/or PD 130908). *Int. J. Radiat. Oncol. Biol. Phys.*, **22**, 545–548.

COLEMAN CN. (1988). Hypoxia in tumors: a paradigm for the approach to biochemical and physiologic heterogeneity. *J. Natl Cancer Inst.*, **80**, 310–317.

DENNIS MF, STRATFORD MRL, WARDMAN P AND WATTS ME. (1985). Cellular uptake of misonidazole and analogues with acidic or basic functions. *Int. J. Radiat. Biol.*, **47**, 629–643.

DISCHE S. (1985). Chemical sensitizers for hypoxic cells: a decade of experience in clinical radiotherapy. *Radiother. Oncol.*, **3**, 97–115.

HORWICH A, HOLLIDAY SB, DEACON JM AND PECKHAM MJ. (1986). A toxicity and pharmacokinetic study in man of the hypoxic cell radiosensitizer RSU 1069. *Br. J. Radiol.*, **59**, 1238–1240.

JENKINS TC, NAYLOR MA, O'NEILL P, THREADGILL MD, COLE S, STRATFORD IJ, ADAMS GE, FIELDEN EM, SUTO MJ AND STIER MA. (1990). Synthesis and evaluation of α-[[(2-Haloethyl)-amino]methyl]-2-nitro-1*H*-imidazole-1-ethanol as prodrugs of α-[(1-Aziridinyl)methyl]-2-nitro-1*H*-imidazole-1-ethanol (RSU 1069) and its analogues which are radiosensitizers and bioreductively activated cytotoxins. *J. Med. Chem.*, **33**, 2603–2610.

OVERGAARD J, SAND HANSEN H, ANDERSEN AP, HJELM-HANSEN M, JØRGENSEN K, SANDBERG E, BERTHELSEN A, HAMMER R AND PEDERSEN M. (1989). Misonidazole combined with split-course radiotherapy in the treatment of invasive carcinoma of larynx and pharynx. Report from the DAHANCA 2 study. *Int. J. Radiat. Oncol. Biol. Phys.*, **16**, 1065–1068.

ROIZIN-TOWLE L, PIRRO JP AND HALL EJ. (1990). Studies with bifunctional bioreductive drugs. *Radiat. Res.*, **124**, S50–S55.

SEKSEK O, HENRY-TOULMÉ N, SUREAU F AND BOLARD J. (1991). SNARF-1 as an intracellular pH indicator in laser microspectrofluorometry: a critical assessment. *Anal. Biochem.*, **193**, 49–54.

SIEMANN DW. (1994). *In vitro* cytotoxicity and chemosensitizing activity of the dual function nitroimidazole RB 6145. *Int. J. Radiat. Oncol. Biol. Phys.*, **29**, 301–306.

TANNOCK IF. (1968). The relation between cell proliferation and the vascular system in a transplanted mouse mammary tumour. *Br. J. Cancer*, **22**, 258–273.

THOMLINSON RH AND GRAY LH. (1955). The histological structure of some human lung cancers and the possible implications for radiotherapy. *Br. J. Cancer*, **9**, 539–545.

VAN ERP PEJ, JANSEN MJJM, DE JONGH GJ, BOEZEMAN JBM AND SCHALKWIJK J. (1991). Radiometric measurement of intracellular pH in cultured human keratinocytes using carboxy-SNARF-1 and flow cytometry. *Cytometry*, **12**, 127–132.

VAUPEL P, SCHLENGER K, KNOOP C AND HÖCKEL M. (1991). Oxygenation of human tumors: evaluation of tissue oxygen distribution in breast cancers by computerized $O_2$ tension measurements. *Cancer Res.*, **51**, 3316–3322.

WALLING J, STRATFORD IJ, ADAMS GE AND STEPHENS MA. (1988). Dual-function radiation sensitizers and bioreductive drugs: factors affecting cellular uptake and sensitizing efficiency in analogues of RSU 1069. *Int. J. Radiat. Biol.*, **53**, 641–649.

WHITMORE GF AND GULYAS S. (1986). Studies on the toxicity of RSU 1069. *Int. J. Radiat. Oncol. Biol. Phys.*, **12**, 1219–1222.

WIKE-HOOLEY JL, HAVEMAN J AND REINHOLD HS. (1984). The relevance of tumor pH to the treatment of malignant disease. *Radiother. Oncol.*, **2**, 343–366.